# Subjective Experiences of Speech and Language Therapy in Patients with Parkinson's Disease: A Pilot Study

**DOI:** 10.1155/2015/839895

**Published:** 2015-07-08

**Authors:** Laura Spurgeon, Carl E. Clarke, Cath Sackley

**Affiliations:** ^1^College of Medical and Dental Sciences, University of Birmingham, Edgbaston, Birmingham B15 2TT, UK; ^2^School of Clinical and Experimental Medicine, College of Medicine and Dental Sciences, University of Birmingham, Edgbaston, Birmingham B15 2TT, UK; ^3^Division of Health and Social Care, Faculty of Life Sciences and Medicine, King's College, 7th Floor Capital House, 42 Weston Street, London SE1 3QD, UK

## Abstract

*Purpose*. Parkinson's disease can produce a range of speech-language pathologies, which may require intervention. While evaluations of speech-language therapy have been undertaken, no work has been undertaken to capture patients' experiences of therapy. This was the aim of the present study. *Methods*. Semistructured interviews, using themes derived from the literature, were conducted with nine Parkinson's disease patients, all of whom had undergone speech-language therapy. Participants' responses were analysed in accordance with Thematic Network Analysis. *Results*. Four themes emerged: *emotional reactions* (frustration, embarrassment, lack of confidence, disappointment, and anxiety); *physical impact* (fatigue, breathing and swallowing, and word production); *practical aspects* (cost of treatment, waiting times, and the actual clinical experience); and *expectations about treatment* (met versus unmet). *Conclusions*. While many benefits of speech-language therapy were reported, several negative issues emerged which could impact adversely on rehabilitation. Parkinson's disease is associated with a range of psychological and physical sequelae, such as fatigue and depression; recognising any individual experiences which could exacerbate the existing condition and incorporating these into treatment planning may improve rehabilitation outcomes.

## 1. Introduction

Parkinson's disease (PD) is a progressive neurological disorder, affecting around 127,000 people in the UK alone [1]. It is caused by a degeneration of neurons in the substantia nigra, leading to reduced dopamine production; this results in the slowing of movements over time [2]. There is currently no cure for PD, with surgical, medical, and therapeutic treatments primarily focusing on slowing the progression of the disease and ameliorating the symptoms. The three key symptoms of PD are tremor at rest, rigidity, and bradykinesia [3], although fatigue, pain, and depression are also common, with the latter occurring in around 40% of PD patients [4]. In addition to the foregoing problems and of particular relevance to the current study is the impact of PD on speech and language. Estimates of the extent of speech-language pathology in PD patients range from 65–74% [5] to 89% [6]. The most common speech-language problems associated with PD include reduced volume (hypophonia), reduced pitch variation, and difficulty articulating syllables (dysarthria); this is particularly impaired when conveying complex thoughts or performing a competing task, such as walking when conducting a conversation [6–8]. Other notable characteristics of PD speech include difficulty initiating speech [7]; tremor [8]; a weak, breathy voice [9]; increased pauses and hesitations; and grammatical and word-finding deficits, which result in difficulty expressing ideas via language [6]. The emotional impact of living with speech-language pathology is well documented in a number of clinical conditions, with depression, negative thoughts, and a reduced quality of life all being acknowledged consequences [10]. Likewise, PD patients' reactions to their speech-language problems include fatigue, social withdrawal and lack of confidence [6], and impaired quality of life [11]; apprehension surrounding social interaction; embarrassment; social withdrawal; a passive approach to conversations; depression; and marital tension [12]. The findings from these studies, together with the suggestion that around 40% of PD patients experience significant depression which may occur independently of any speech-language pathology [4], clearly identify psychosocial problems as a salient correlate of PD.

Despite the prevalence of speech-language pathology in PD and its psychological ramifications, therapy provision in the UK remains limited, with fewer than 40% of patients receiving intervention [13]. Currently, the Royal College of Physicians [14] recommends two types of speech-language therapy: generic National Health Service (NHS) speech-language programmes and PD-specific Lee Silverman Voice Treatment (LSVT). While the approaches differ in their origins and their method of delivery, both approaches aim to improve voice loudness and control, in order to enable more effective daily functioning. Because of its generic nature, attempts to ascertain the efficacy of NHS speech-language therapy have been hampered by a number of methodological issues; these include a lack of clarity about and control over the specifics of the style, intensity, and duration of the intervention [15]. However, there is some evidence that since the focus of rehabilitation is on the impaired attention-to-effort and reduced monitoring of vocal output that characterise PD speech-language pathology, then these generic NHS interventions may be successful in improving aspects of speech production [7, 16]. In contrast, the LSVT programme was developed specifically for PD patients and is an extremely intensive and structured therapeutic package, focusing on a single speech feature at a time [17, 18]. Evaluations of the programme report typically positive (if qualified) clinical outcomes [18–20] though there is some evidence that, irrespective of the intervention, it is the intensity of the programme that is the effective element, rather than the specifics of the method itself [7].

If this contention has substance, then it has wider implications. Firstly, the required level of input and activity may be counterproductive for other symptoms of PD, even where there are detectable communication gains; in this sense, any benefits for communication may be offset by increased fatigue and associated depression. Secondly, evidence from other clinical contexts, such as stroke rehabilitation, has highlighted the improvement in clinical outcomes when the patient's subjective experience is factored into treatment interventions [21, 22]. And, thirdly, it is well established that the success of any health intervention depends heavily on a range of patient-centred psychological factors, such as health beliefs, illness behaviours, and motivation [23]. If these findings have application to PD, then it would be reasonable to conclude that the patient's experience of speech-language intervention could have a bearing on rehabilitation outcomes and intervention decisions. It is surprising, therefore, that there is a paucity of research that has investigated the experiential angle of speech-language therapy among PD patients. This omission may be highly significant in the context of PD, accompanied as it is by high rates of negative affect, psychosocial difficulties, and exhaustion, all of which could adversely affect and be affected by speech-language pathology and therapy. It follows that if speech-language therapy is to be optimised, it would seem important to ascertain PD patients' experiences of intervention, in order that the care package can be better managed and targeted to individual needs, a position consistent with NICE guidelines [24]. To date, no work of this type has been undertaken, and this, therefore, constituted the basis for the current small-scale study. More specifically, the aim was to establish PD patients' experiences of speech-language therapy in order to establish how these could potentially impact on therapy decisions and outcomes.

## 2. Methods

### 2.1. Design

The principal method used here was a qualitative interview approach designed to capture experiential, phenomenological data. Interviews are a useful tool for gathering a substantial quantity of rich subjective data, by allowing respondents the freedom to talk openly and, if desired, at length about their feelings, opinions, and experience of a particular topic [25]. The study used a small sample of nine participants, in line with Crouch and McKenzie's recommendation [26], to “enhance the validity of fine-grained, in-depth inquiry in naturalistic settings” (p483). Because of this, the study is of necessity small-scale; no hypotheses could be tested; and the findings cannot be generalised. However, such a small in-depth study provides richness of data that can complement other data-types and act as a first-stage activity for further data-collection.

Data were gathered via semistructured telephone interviews without time limit. Telephone interviews were chosen for two reasons: firstly, the participants were distributed across the whole of the UK, creating practical problems for access within the research time-frame; and, secondly, telephone interviews allow the discussion of sensitive issues that might be difficult to broach face-to-face [27]. As the validity of telephone interviews is considered to be comparable to that of face-to-face interviews [28], this approach was not considered to compromise the design. The interview narratives were distilled using Attride-Stirling's Thematic Network Analysis [29], which elicits common core themes that ran through patients' subjective experiences.

### 2.2. Sample

A convenience sample of nine PD patients was recruited following a request posted on a number of online PD support-group websites. All the participants had confirmed idiopathic PD and had developed speech difficulties within the first year after diagnosis. None had any physical or mental health comorbidities or were on medication for any clinical condition other than PD; all had received and completed either NHS speech-language therapy or LSVT within the 3 years prior to the study. Referral for speech-language therapy was via the GP or consultant. The NHS therapy comprised around 6–8 × 60-minute sessions over a two-month period, while the LSVT intervention comprised the standard 16 × 60-minute sessions over 4 weeks. Participants ranged in age from 54 to 78 and comprised seven male and two female respondents (for sample details see Table 1). All volunteers were provided with a letter outlining the nature and purpose of the study and all signed a consent form in advance of the interview.

### 2.3. Ethics

Ethical approval for this pilot study was given National Research Ethics Service (NRES) approval (reference: 11/WM/0343).

### 2.4. Procedure

To select the topics for inclusion in the semistructured interviews, a comprehensive literature review was undertaken, using the following search engines: Medline, PsychINFO, CINAHL, Google Scholar, AMED, Cochrane Library, and ASSIA. Despite the use of a wide variety of key words, very little PD-specific research exists in relation to the experience of speech-language therapy, and therefore the search was extended to include other clinical groups undergoing speech-language therapy. The interview topics/questions can be found in the following section. Participants were asked the ten open-ended questions, derived from the literature search; these included prompts, used, for example, if a participant felt that they were unable to articulate an idea. The prompts typically involved asking the respondent to expand or develop something they had already said or asking for specific examples or details about an idea. Participants' responses were recorded in note-form throughout the interview. The first author (Laura Spurgeon) undertook all the interviews to maintain consistency of interview technique.

*Semistructured Interview Topics.* The topics were as follows:

1. *Clinical background:* when were you diagnosed with Parkinson's disease?
2. *Nature of speech problems:* what was the nature of your speech problems? That is, what speech impairments you had; approximate date of when your speech problems occurred in the course of your Parkinson's disease; and so forth.
3. *Impact of speech problems:* what impact did the speech problems have on your life?
4. *Speech-language therapy:* what treatment did you receive? For example, standard NHS provision, Lee Silverman Voice Treatment, number of sessions, what you did in the sessions, and homework.
5. *Reactions to speech-language therapy:* how did you feel about the speech therapy at the time? Was it something you were keen/reluctant to do?
6. *Impact of speech-language therapy:* what impact has the speech therapy had on your life? Please include positive and negative aspects, IF applicable.
7. *Experience of speech-language therapy:* how would you describe the overall experience of your speech therapy?
8. *Wider impact of speech-language therapy:* did the speech therapy have any other effects on you? For example, on your tiredness levels, emotional wellbeing, other aspects of your Parkinson's, and social/occupational life.
9. *Overall assessment of speech-language therapy:* what would you say to someone else considering speech and language therapy?
10. *Other issues:* are there any other comments you wish to make about your speech therapy?

### 2.5. Data Analysis

The data from the interviews were analysed using thematic analysis, a technique for distilling narrative data in order to identify common themes [30]. Thematic analysis is suitable for a range of research topics, may be used with data that have been derived from a variety of sources, and is appropriate for large or small databases [30]. Generally, thematic analysis involves 6 stages: familiarisation with the data; coding the data; searching for themes; reviewing themes; naming the themes; and writing up the findings [30]. Many thematic analysis techniques are available, but the method selected here was Attride-Stirling's Thematic Network Analysis (TNA) [29]. This method provides a sophisticated and robust tool for analysing qualitative data, producing three hierarchical levels of information: basic themes (the information that is derived from the narrative/text data); organising themes (clusters of similar basic themes); and global themes (overarching categories that include all the basic and organising themes). These levels of information are presented in a representational network, described by Attride-Stirling as “web-like illustrations that summarise the main themes constituting a piece of text” [29, p1]. Attride-Stirling further notes that the thematic network is not in itself analysis, but simply a tool in the process; interpretation of the themes and networks produced is still required by the researcher. The TNA technique generates a vast array of narrative information and the process of data-reduction is lengthy; therefore, to illustrate the method here, a highly simplified stepwise example of the process is provided as follows (see also Table 2).

**Step 1 (coding the narrative material).**

The first stage in reducing the narrative data involved breaking it down into manageable segments using a coding framework. There are many ways to do this, but, here, key words in the participants' statements were first identified and grouped semantically using Roget's Thesaurus. With reference to Table 2, a small selection of narrative statements can be found in column 1, with the relevant semantic code in column 2. These narrative statements constitute the basic themes.

**Step 2 (identifying themes).**

The purpose of this stage is to represent the coded narrative text succinctly. From the semantic codes, common themes were initially abstracted by identifying the key common issues identified in the coded text. These were later refined to achieve maximum specificity (to avoid repetition) and maximum breadth (to ensure that similar ideas were contained within a theme). These themes are presented in column 3 in Table 2 and comprise the organising themes.

**Step 3 (constructing the networks).**

The themes must be then arranged into similar groupings to provide the thematic networks; these may include antonyms. From the organising themes, superordinate global themes which encapsulate the essence of the organising themes can be deduced; these are presented in column 4, Table 2.

In order to embed as much rigour as possible in the data-distillation process, it is recommended that the thematic analysis be conducted by at least two people, working independently. Here, the data reduction was conducted separately by three psychologists; all were independent of the study so that any* a priori* assumptions about the themes could be minimised. The level of agreement between the initial categorisation was assessed using a Thesaurus to determine the semantic similarity of the themes; the number of similar themes that all three researchers identified was calculated as a percentage of the total number of themes generated. This degree of objectivity is not a requirement of the TNA process but was considered here to add to the validity of the process and outcome. There was a high level of initial semantic agreement between the three researchers on both the organising and global themes (>70%). Further discussion between them, which involved expanding on their individual interpretations of the raw data and explanations of the initial groupings, raised the level of agreement to >85%. Again, this degree of rigour is not a prerequisite of the TNA process.

## 3. Results

A simplified example of the initial analysis is presented in Table 2.

**Figure 1:**
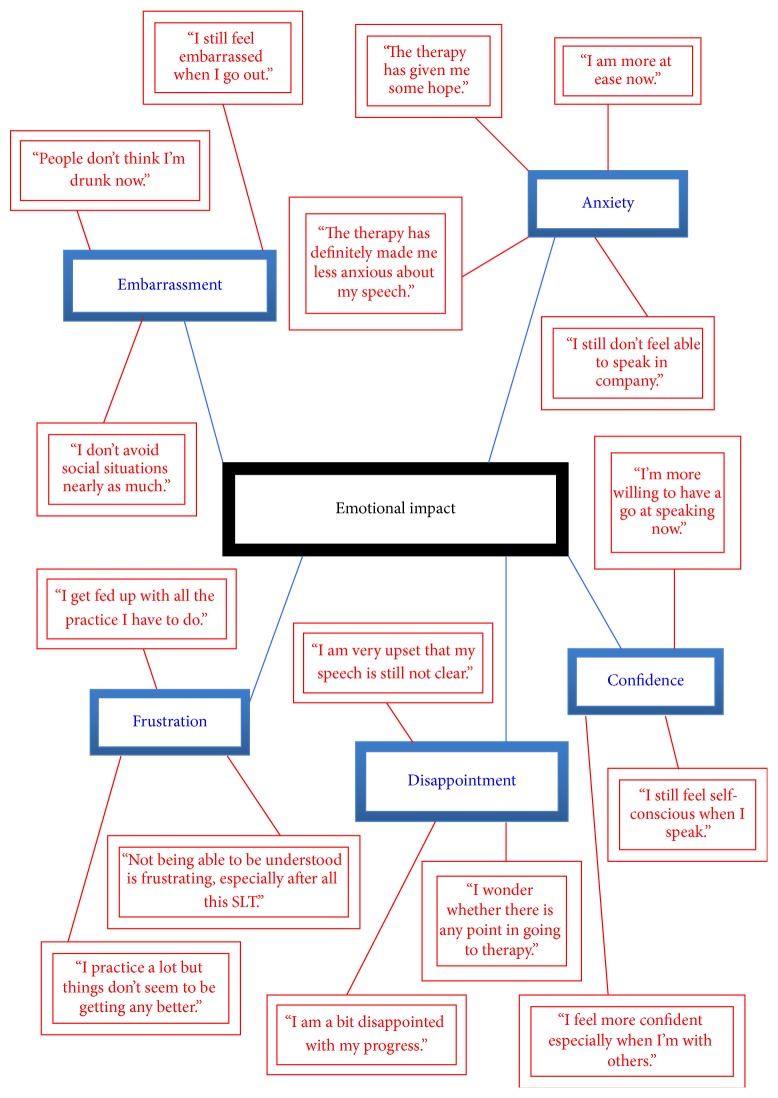
Thematic Network Analysis: emotional impact.

**Figure 2:**
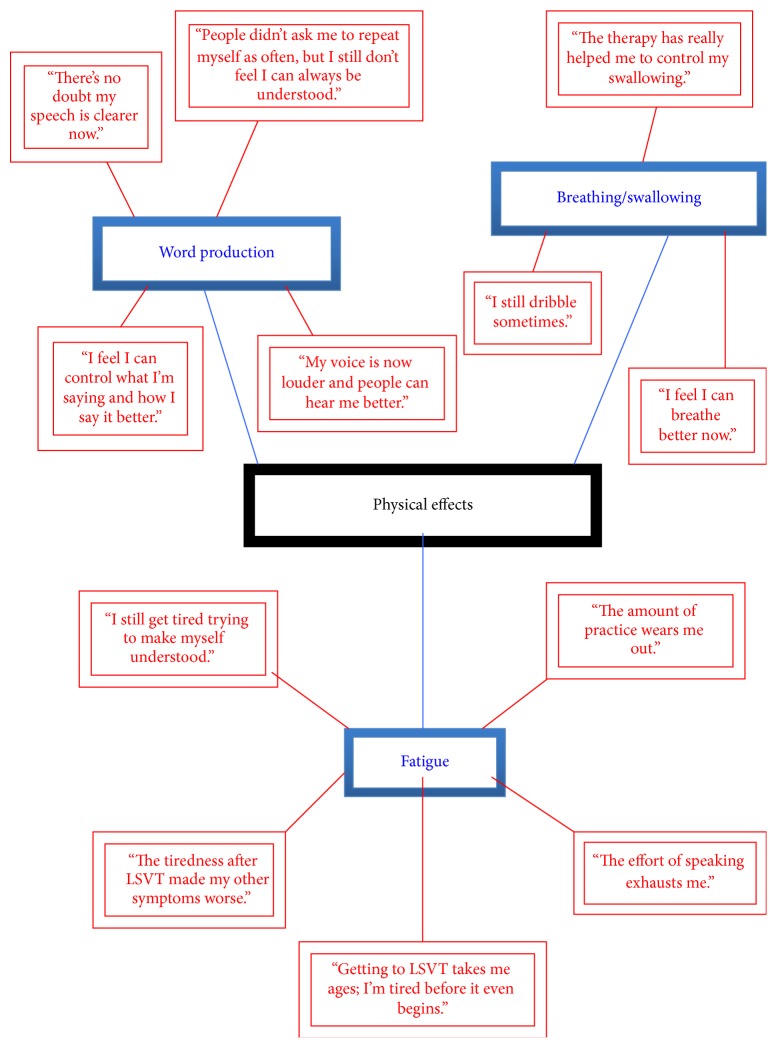
Thematic Network Analysis: physical effects.

**Figure 3:**
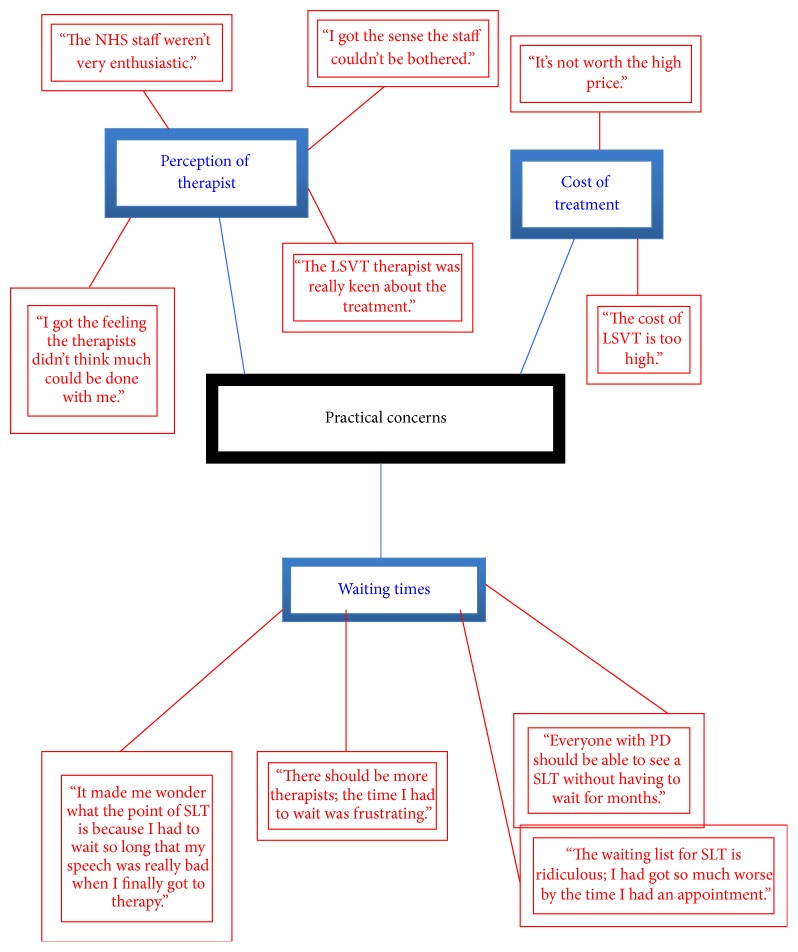
Thematic Network Analysis: practical concerns.

**Figure 4:**
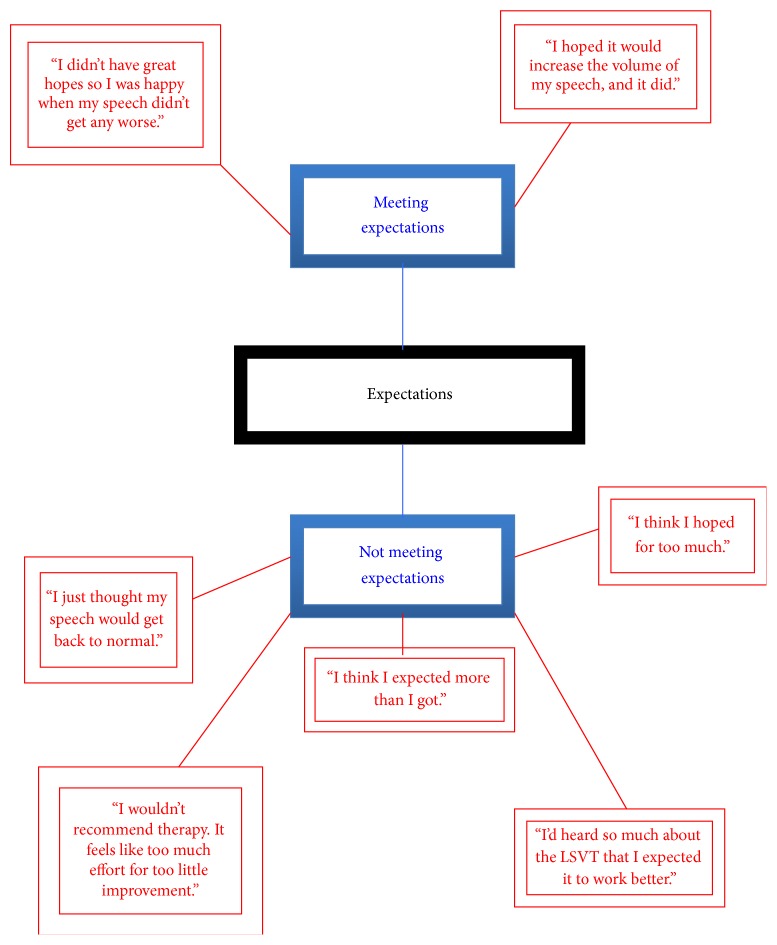
Thematic Network Analysis: expectations.

The themes that emerged from the TNA were named as follows:* emotional impact*;* practical concerns*;* physical effects*; and* expectations*. Highly simplified illustrative networks are presented in Figures 1–4 (basic themes: red; organising themes: blue; global themes: black). It should be noted that the basic themes have been abbreviated and extracted from the context in which they originally occurred. Therefore, while some basic themes may not appear in the figures to accord with the associated organising theme, they are consistent with the detailed interview discussion from which they emerged. Further, because of the significant amount of raw data that constituted the basic themes, only a selection of illustrative comments has been included, in order to demonstrate the TNA process.

## 4. Discussion

This small-scale pilot study involved collecting and thematically analysing qualitative data from semistructured interviews with PD patients who had undergone speech-language therapy. Through this process, four key themes emerge, each of which will be discussed in turn. Illustrative examples of themes are provided from the raw data and may not be the same as the examples given in the figures above.

The first theme to emerge was labelled “*emotional impact of speech therapy*” and, from the number and nature of comments supplied, was the biggest issue for the interviewees. The emotional reactions included continued frustration, embarrassment, (lack of) confidence, disappointment, and anxiety. The responses tended to be bound up with the persistent speech problems experienced after speech-language therapy and reflected respondents' assumptions that the associated negative affective reactions would have abated after therapy. The frustration, strain, and irritation of trying to produce words that were often still unintelligible to others caused a profound dissatisfaction that sometimes led to depression and social isolation (“after all that hard work, not being able to say what I wanted or not being understood by others when I did speak was infuriating – so much so that I often chose to stay in”). Having to repeat themselves frequently was aggravating and in some cases led to an increase in domestic arguments (“I definitely felt the arguments with my wife increased as my speech and language difficulties worsened; I think it was because we thought I would be doing better by now”). Five interviewees referred to the continuing embarrassment in social situations when they tried to speak (“people still think I'm drunk because I slur my words”), although seven participants commented on an improvement in general self-esteem and confidence consequent upon therapy. The dominant emotional reaction was anxiety, with a concern that speech would probably never get back to anything approaching normality expressed by all nine interviewees (“it all feels a bit hopeless; I'm never going to be able to speak as I want to”). Seven interviewees noted that anxiety temporarily reduced immediately following therapy (“I felt more confident to go out and be with friends after therapy”; “I felt quite encouraged after therapy sessions”), but any immediate positive emotional impact of therapy was not always sustained overall. While this theme clearly illustrates the emotional reactions following therapy, there was clear lack of accord between what the respondents had hoped to achieve and what was actually achieved. Consequently, this theme is integrally linked with the theme of “expectations.” The issues raised under this global theme resonate with research that has been conducted on patient experiences of speech problems generally. For example, social withdrawal and lack of confidence have been reported [6, 12, 31], along with embarrassment [12] and disappointment/depression [10, 12]. While the foregoing studies focused on the experience of living with speech impairments, the participants in the current study reported comparable reactions to their persistent speech and language problems after therapy. In other words, the experience of speech-language therapy had not apparently ameliorated the generally negative affect caused by their speech problems. Since PD is frequently accompanied by depression [4], the impact of speech-language therapy on the patient's emotional state, and vice versa, is of considerable relevance in the management of speech-language pathology in this clinical group. Undoubtedly, any PD-associated depression is likely to affect the patient's motivation to undertake therapy, their commitment to it, and their appraisal of the process; this may affect therapy outcomes, which in turn would exacerbate the patient's emotional state, leading to a downward spiral of negative affect. Therefore, mid-to-longer term monitoring of patients' psychological state might be indicated, as well as ensuring patients receive adequate advice, information, and support before and after therapy.

The second theme was labelled “*physical effects of therapy*” and included issues relating to fatigue, breathing, swallowing, and word production. The fatigue derived from the exertion involved word production, the amount of intersession practice required, and the effort required to get to therapy sessions. Generally, the lack of widely available speech-language therapy of any kind in the UK means that many patients cannot easily access therapy and therefore makes the current official recommendations [14, 24] difficult to implement [13]. Of the nine interviewees, four reported that the actual therapy sessions had increased their overall fatigue levels, with two participants noting that, as a consequence, their other PD symptoms had deteriorated (“the sessions are so demanding I felt more exhausted than usual afterwards”). Despite the negative impact of fatigue, all nine interviewees noted that therapy of both sorts had improved some aspects of their speech, especially volume and reduction in stuttering and pitch (“people didn't ask to me to repeat myself as much, so my articulation must have improved a lot”; “my wife commented on how much I'd improved”). Four of the nine interviewees reported a general improvement in swallowing and breathing following therapy. Together, the predominant reaction to therapy was an acknowledgement of the overall benefit to speech production; this is consistent with other research which has reported the positive impact of speech-language therapy on word production in PD patients [18, 32]. Whilst acknowledging the beneficial effects of therapy on speech, the additional tiredness, reported by these respondents whose condition is already characterised by high levels of fatigue, should be a consideration in treatment planning and management.

The practical aspects of therapy uptake were a significant theme for many respondents and included the cost of treatment, waiting times, and the actual clinical experience. The cost of treatment was an issue for the two participants who had received LSVT only and was linked with some doubt that the outcomes were worth the price (“the benefits I got from LSVT were not worth the price I paid”). Generally, there was concern about waiting list times, which may reflect the overall shortfall in speech-language therapists nationally and/or a primary focus on other patient groups (“I had to wait so long for my first session that my speech had got so bad and I wondered whether it was worth going”). Three interviewees referred to their perceptions that some speech-language therapists lacked apparent commitment to the process, while one interviewee noted the therapist's enthusiasm. An explanation for these conflicting comments may be traced back to the type of speech-language therapy received by these interviewees, with perceived lack of commitment being associated with NHS speech-language therapists, and enthusiasm with LSVT. The comments may reflect some dissonance reduction among the LSVT therapists; given that the LSVT requires additional training, it is likely that those therapists who undertake this are highly committed both to PD and to this intervention. While these findings should not be assumed to reflect the attitudes of speech-language therapists more generally, it is nevertheless important to note that the reactions of the therapists may influence both the patient's outcome and their perceptions of the efficacy of the intervention; this may be worthy of further study.

Met versus unmet expectations about treatment outcome characterised the fourth theme and suggested a possible realistic versus unrealistic dimension. Those participants whose expectations of speech-language therapy were high were typically disappointed both with the clinical outcomes and the maintenance of any benefits over time (“I didn't make the progress I had hoped for, and even with lots of practice, I haven't been able to keep up the improvements”; “I'd heard so much about the LSVT that I thought my speech would go back to normal afterwards”). Conversely, those patients who expected less, expressed greater satisfaction with the process and the results (“I just hoped that it would help me speak a bit louder and it did, so I'm OK with that”). No objective measure of therapy outcomes was included here, and therefore it is impossible to assess whether participants' experience is related to any actual voice/language improvement. Clearly, a number of human factors can impact on subjective perceptions; for example, dissonance reduction may have affected some participants, with high investment of effort or money potentially influencing assessments, irrespective of outcome. Similarly, PD patients assuming that speech-language therapy will restore their voice and speech to normal are likely to be dissatisfied with anything less. Therefore, a clear statement of goals, together with proactive management of patient expectations, are critical to sustaining patient engagement in the therapeutic process generally [23] and to speech-language therapy specifically [33]. It would seem important, therefore, to make explicit to PD patients, at the outset, what the realistic targets are and the extent to which these accord with the patient's own expectations, not only to promote patient commitment and compliance, but also to avoid further depression that might result from nonattainment of personal goals. The management of any conflict between expectations and realistic outcomes should therefore be an essential part of the treatment programme.

Together, the themes emerging from this study complement the findings that have been documented elsewhere. The majority of participants acknowledged the benefits of speech-language therapy to their general wellbeing, quality of life, and speech; however, this was not verified by any psychometric measurement, nor by any objective assessment of therapy outcomes. Nonetheless, these positive outcomes were tempered to an extent by a range of negative responses that have the potential to adversely influence patients' psychological adjustment, their preparedness to engage and comply with therapy, and the success of any intervention. In other words, there was a clear perceived benefit of therapy, but an equally clearly perceived cost. If the effective ingredient in speech-language therapy for PD patients is intensity [7], it is possible that the impact of this may exacerbate existing fatigue and influence expectations. Therefore, acknowledging psychological reactions both to the disease and to therapy and accommodating these in the care plan may have a positive impact on both clinical and psychosocial outcomes. At the very least, these findings suggest three clear recommendations: firstly, the need to ensure that PD patients are adequately informed, monitored, and supported throughout the speech-language process; secondly, that therapists and other health-care professionals are aware of the potential adverse impact of speech-language therapy on other aspects of the condition, in particular, depression and fatigue; and, thirdly, that the patient's experience is incorporated into the management and delivery of speech-language rehabilitation.

This was a small pilot study and as such has inevitably been limited by sampling issues, although the nature and numbers of the participants involved were consistent with recommended guidelines. Furthermore, the interviews relied on memories that were up to three years old and therefore could have been unreliable; the motives of those who volunteered to take part may also have influenced the findings, while the differences between the speech-language approaches may also have impacted aspects of participants' experiences. Nonetheless, it has provided some valuable and completely original data, which demonstrate a number of common issues that affect PD patients undergoing speech-language therapy, namely, its emotional impact, physical effects, practical concerns, and expectations. Together, these types of experience may exacerbate the existing core symptoms and sequelae of PD, which in turn have the potential to create a downward trajectory of the clinical condition. If these findings could be replicated by wide-scale survey data, they would point the way towards a number of possible modifications to current speech-language provision for PD patients: firstly, that the management and provision of speech-language therapy for PD patients are informed by the patient's experiences before, during, and after therapy and, in this way, could optimise rehabilitation outcomes; secondly, ensuring that speech-language therapists, as well as patients, are fully informed about the potentially adverse physical and psychological sequelae of intervention for PD patients; and, thirdly, that health care professionals attempt to ensure that patients' expectations of therapy are realistic. In this way, speech rehabilitation outcomes could be optimized, with all the associated psychosocial benefits.

## Figures and Tables

**Table 1:** Sample details.

Participant number	Age	Gender	Years since diagnosis	Nature of speech problem	SLT received
1	76	Female	18	Hoarse, breathy, and monotonous	NHS
2	54	Male	1	Hoarse, monotonous, and hesitant	NHS
3	67	Male	3	Breathy and slurred	NHS
4	59	Female	9	Hoarse, breathy, and slurred	NHS
5	66	Male	12	Monotonous and slurred	NHS & LSVT
6	74	Male	7	Breathy, monotonous, and often unintelligible	NHS
7	59	Male	6	Quiet and hoarse monotonous	NHS & LSVT
8	67	Male	7	Hesitant, quiet, monotonous, and stuttering	LSVT
9	74	Male	8	Quiet, slurred, and monotonous	LSVT

**Table 2:** A simplified illustration of the preliminary development stages of the thematic analysis.

Narrative statement (Basic themes)	Semantic code	Themes identified (Organising themes)	Global theme
I worry that my speech will never improve	Anxiety	Anxiety	Emotional impact

I am not as self-conscious when speaking	Self-consciousness	Confidence	Emotional impact

I feel irritated by not being able to say what I want to, especially after all this therapy	Irritation	Frustration	Emotional impact

I feel very upset that I still cannot speak well enough to be understood	Concern	Disappointment	Emotional impact

I feel that, no matter how hard I try, my speech is no better	Disappointment	Disappointment	Emotional impact

The therapy is exhausting at times, and I often feel worse afterwards	Tiredness	Fatigue	Physical impact

I am not nearly as depressed about my speech	Depression	Disappointment	Emotional impact

I do not feel that therapy has done what I had hoped for	Thwarted expectations	Expectations	Expectations

The increased volume in my speech is noticeable and has made a big difference	Satisfaction	Expectations	Expectations

I am always worn out after a therapy session	Tiredness	Fatigue	Physical impact

I get fed up with the amount of practice I have to do	Irritation	Frustration	Emotional impact

The cost of LSVT is too high	Cost	Financial commitment	Practical concerns

I waited ages to get therapy and my speech went downhill during that time	Wait times	Waiting times	Practical concerns
