# Corrigendum to “Subjective Experiences of Speech and Language Therapy in Patients with Parkinson's Disease: A Pilot Study”

**DOI:** 10.1155/2016/1654282

**Published:** 2016-08-17

**Authors:** Laura Spurgeon, Carl E. Clarke, Cath Sackley

**Affiliations:** ^1^College of Medical and Dental Sciences, University of Birmingham, Edgbaston, Birmingham B15 2TT, UK; ^2^School of Clinical and Experimental Medicine, College of Medicine and Dental Sciences, University of Birmingham, Edgbaston, Birmingham B15 2TT, UK; ^3^Division of Health and Social Care, Faculty of Life Sciences and Medicine, King's College, 7th Floor Capital House, 42 Weston Street, London SE1 3QD, UK

In the article titled “Subjective Experiences of Speech and Language Therapy in Patients with Parkinson's Disease: A Pilot Study”, [1] the “Acknowledgments” section was missing, and should be added as follows.
